# A Novel Investigation for Early Sex Determination in Alive Adult European Seabass (*Dicentrarchus labrax*) Using *cyp19a1a*, *dmrt1a*, and *dmrt1b* Genes Expression in Tail Fin tissues

**DOI:** 10.1007/s10126-024-10313-z

**Published:** 2024-04-23

**Authors:** Samy Y. El-Zaeem, Amr El-Hanafy, Alaa A. El-Dahhar, Ayaat M. Elmaghraby, Sara F. Ghanem, Amany M. Hendy

**Affiliations:** 1 Animal and Fish Production Department, Faculty of Agriculture - Saba-Basha, Alexandria University, Alexandria, Egypt; 2 Nucleic Acids Research Department, Genetic Engineering and Biotechnology Research Institute (GEBRI), City of Scientific Research and Technological Applications, Alexandria, Egypt; 3 National Institute of Oceanography and Fisheries, NIOF, Alexandria, Egypt; 4 Faculty of Health Sciences Technology, Borg Al-Arab Technological University, Alexandria, Egypt

**Keywords:** *Dicentrarchus labrax*, Sex determination-related genes, Tail fin, Discriminant analysis, *cyp19a1a*

## Abstract

This study is the first investigation for using sex-related gene expression in tail fin tissues of seabass as early sex determination without killing the fish. The European seabass (*Dicentrarchus labrax*) is gonochoristic and lacks distinguishable sex chromosomes, so, sex determination is referred to molecular actions for some sex-related genes on autosomal chromosomes which are well known such as *cyp19a1a*, *dmrt1a*, and *dmrt1b* genes which play crucial role in gonads development and sex differentiation. *cyp19a1a* is expressed highly in females for ovarian development and *dmrt1a* and *dmrt1b* are for testis development in males. In this study, we evaluated the difference in the gene expression levels of studied genes by qPCR in tail fins and gonads. We then performed discriminant analysis (DA) using morphometric traits and studied gene expression parameters as predictor tools for fish sex. The results revealed that *cyp19a1a* gene expression was significantly higher in future females’ gonads and tail fins (*p* ≥ 0.05). Statistically, *cyp19a1a* gene expression was the best parameter to discriminate sex even the hit rate of any other variable by itself could not correctly classify 100% of the fish sex except when it was used in combination with *cyp19a1a*. In contrast, *Dmrt1a* gene expression was higher in males than females but there were difficulties in analyzing *dmrt1a* and *dmrt1b* expressions in the tail because levels were low. So, it could be used in future research to differentiate and determine the sex of adult fish using the *cyp19a1a* gene expression marker without killing or sacrificing fish.

## Introduction

Aquaculture has made a significant contribution to global food production, particularly in providing a sustainable source of animal proteins. According to the Food and Agriculture Organization (FAO 2019), the total aquaculture production worldwide reached approximately 85.4 million tons. In Egypt, which boasts the largest aquaculture industry in Africa, aquaculture plays a pivotal role in the provision of fish, accounting for a total production of around 1.8 million tons; however, 11.52% of the total production is attributed to the European seabass (*Dicentrarchus labrax*), which is the first marine species cultivated (Kaleem and Bio Singou Sabi 2021).

The European seabass (*D. labrax*) is a member of the Moronidae family of Teleosts, which is closely related to the Serranidae (groupers) family and includes numerous hermaphroditic species. It is worth noting that *D. labrax* is gonochoristic and lacks readily distinguishable morphological secondary sexual features or sex chromosomes (Piferrer et al. 2005; Vandeputte et al. 2007). The initial histological signs of ovarian and testicular differentiation become evident when male and female fish attain a standard length (SL) of approximately 79–90 mm and 83–95 mm, respectively (occurring around 150–200 days post fertilization (dpf)) (Saillant et al. 2003). Economically, the aquaculture sector is interested in the monosex breeding of female cohorts because females take longer to mature and grow than males, which results in more weight gain (Wootton and Smith 2014). Hence, investigations for the identification of sex-determining genes and the elucidation of their functioning in fish are expected to provide a conceptual framework to inform the development of strategies for sex control in breeding programs (Long et al. 2020; Chen et al. 2022).

Genes responsible for sex determination in various fish species reveal a wide spectrum of diversity. So, understanding the mechanisms behind sex determination in the majority of fish species used in aquaculture is challenging due to the intricate and varied nature of these processes (Lin et al. 2017). In the case of seabass, several genes involved in sex determination and differentiation have been identified and their expression profiles have been examined. These genes include *cyp19a1a* (Dalla Valle et al. 2002; Blázquez et al. 2008), *cyp19a1b* (Blázquez and Piferrer 2004; Blázquez et al. 2008), *amh* (Halm et al. 2007), *dax1* (Martins et al. 2007), estrogen receptors (*ers*) (Halm et al. 2004; Blázquez et al. 2008), androgen receptor *b* (*arb*), (Blázquez and Piferrer 2005), and *cyp11b* (Socorro et al. 2007).

The cytochrome P450 family 19 subfamily A (*cyp19a*), also referred to as *cyp19a1*, *cyp19a1a*, and ovarian aromatase (Guiguen et al. 2010), demonstrates a distinct pattern of expression. Where, *cyp19a1a* exhibits a high level of expression in the ovary, with comparatively lower expression in the testis and brain (Dalla Valle et al. 2002). Aromatase, well known for its pivotal role in sex differentiation in fish, is presumed to play a significant role in the process of sex determination in seabass (Blázquez and Piferrer 2004). In this context, research by Blázquez et al. (2009) has demonstrated that *cyp19a1a* expression is significantly elevated in individuals destined to become females, establishing it as a robust molecular marker for predicting future ovarian differentiation in seabass.

The double sex and mab-3-related transcription factor 1 (*Dmrt1*) gene belongs to a gene family characterized by a zinc-finger-like DNA-binding motif known as the DM domain which was identified in both invertebrate and vertebrate species (Guo et al. 2005). Dmrt1 is notably expressed in the gonads, especially in the testes, of various species such as tilapia (*Oreochromis niloticus*), rainbow trout (*Onchorynchus mykiss*), medaka (*Oryzias latipes*), and fugu (*Takifugu rubripes*) (Guan et al. 2000; Marchand et al. 2000; Brunner et al. 2001; Yamaguchi et al. 2006). In the case of the European sea bass (*D. labrax*), a single gene encodes two distinct dmrt1 transcripts, *dmrt1a* and *dmrt1b*, both of which are specifically expressed in males (Deloffre et al. 2009).

Selective breeding programs depend on the identification of secondary sexual features or identifiable sex chromosomes which are lacking in European seabass, although identified morphological features are a crucial prerequisite to achieving results for breeding programs as well as for safeguarding biodiversity, so using *cyp19a1a*, *dmrt1a*, and *dmrt1b* expressions in sexually differentiated adult fish have already been shown to be sexually dimorphic in several investigations and their expression levels might potentially be a good indicator of the phenotypic sex. So, the main objective of this study is to investigate the expression of *cyp19a1a*, *dmrt1a*, and *dmrt1b* genes in gonads of 1.5-year-old sexually differentiated seabass males and females in addition to their expression in tail fins (caudal) tissues to assess their expression as molecular markers could be used in future research to differentiate and determine the sex of adult fish without kill or sacrificing fish.

## Materials and Methods

### Sample Collection

The experimental fish used in the present study were 24 adult fish (12 males and 12 females; age 1.5 years, after the gonadal developmental period (540 days post fertilization, dpf)); individuals were collected from the fish hatchery Kilo 21, Alexandria, Egypt. In summary, during the sampling process, the fish were assessed for various parameters, including body weight (BW), total length (TL), and standard length (SL). They were then sorted into two distinct groups based on size: one group comprised the smaller fish with an average SL of 18.5 cm (referred to as the male-dominant group), and the other group consisted of larger fish with an average SL of 20.2 cm (referred to as the female-dominant group). This classification was made considering the well-established association in seabass between somatic growth and phenotypic sex from the early stages of development (Vandeputte et al. 2007). Subsequently, the fish gonads and tail or caudal fins from each individual were carefully dissected. The gonads were examined by the microscope to determine their respective sexes. After this, the collected tissues were individually separated in sterilized tube and quickly frozen in liquid nitrogen and stored at – 80 °C for later analysis. All animal maintenance and handling procedures followed the recommendations of the Institutional Animal Care Use Committee, Alexandria University, Egypt (Alex-IACUC) review report AU: 14/20/11/01/2/9.

### Fulton’s Condition Factor (*K*)

The Fulton’s condition factor (*K*) serves as an indicator of fish well-being and offers valuable insights into aspects such as growth, age, reproductive status, nutritional health, and overall welfare. This factor is determined through the following formula: *K* = (100 × BW)/TL^3^, where BW represents body weight (in grams) and TL corresponds to total length (in centimeters) (Gonzalez-Martinez et al. 2021).

### RNA Extraction

Total RNA was isolated from the ovary, testis, and apical parts of tail fin clip tissues using the Genozol Tri RNA Kit (Geneaid) according to the manufacturer protocol. The RNA yield quality and concentrations were checked by Nanodrop spectrophotometer (BioDrop, England). The normalization of RNA sample concentration was performed to be 50 ng for each sample.

### RT-PCR Reaction for Genes of Interest

Topreal™ One-step RT q-PCR Kit (*SYBER Green with low ROX*) (enzynomics) is used to perform RT and q-PCR reactions according to the manufacturer procedure for expression analysis. *18s* rRNA gene is used as a reference gene. The reaction conditions were as follows: holding at 45 °C for 30 min, PCR reaction was initiated at 95 °C for 10 min, followed by 95 °C for 5 s then annealing step for 30 s for various primers *cyp19a1a*, *dmrt1a*, *dmrt1b*, and *18s* rRNA genes. The reaction was repeated for 50 cycles (Table 1). The specificity of the real-time PCR amplification was confirmed through a melting curve analysis, which confirmed that only a single PCR product of the intended size was selectively amplified. The cycle threshold (Ct) was determined for each individual replicate, and the ultimate values were derived from the average of two replicates for each sample. To normalize the expression values of the genes under investigation, the expression values of the reference gene *18s* rRNA were used.

**Table 1:** The primer sequences and their features employed for amplifying the genes under investigation are provided

**Name**	**Primer sequence**	**Amplicon size (bp)**	**Annealing**	**Reference**
***cyp19a1a*-F**	AGACAGCAGCCCAGGAGTTG	106	60 °C	Blázquez et al. (2008)
***cyp19a1a*-R**	TGCAGTGAAGTTGATGTCCAGTT			
***dmrt1a*- F**	CACCCACCAGTACCTCCACTTCCT	276	50 °C	Deloffre et al. (2009)
***dmrt1a*-R**	GCTGCTGGGTAGTAAGAATGC			
***dmrt1b*-F**	AGACAGGAGATGGTGCCAGATAAG	121		
***dmrt1b*-R**	GCTGCTGGGTAGTAAGAATGC			
***18s*** **rRNA** *-****F***	TCAAGAACGAAAGTCGGAGG	110	60 °C	
***18s*** **rRNA** ***R****-*	GGACATCTAAGGGCATCACA			

The antisense primer was shared between the two *dmrt1* genes

*cyp19a1a* the cytochrome P450 family 19 subfamily A, *dmrt1a* and *dmrt1b* the double sex and mab-3 related transcription factor 1 transcripts

The analysis of gene expression for the genes under examination was conducted using the $2^{-\Delta\Delta {C_t}}$ method, as outlined in the following equation (Livak and Schmittgen 2001):

- Fold difference = $$2^{-\Delta\Delta {C_t}}$$
- Δ*C*_t sample_ – Δ *C*_t calibrator_ = ΔΔ *C*_t_
- *C*_t GOI_ s − *C*_t norm_ s = Δ *C*_t sample_
- *C*_t GOI_ c − *C*_t norm_ c = Δ *C*_t calibrator_

The ΔΔCt method stands as a widely adopted approach for comparing outcomes between experimental samples and employs both a calibrator (e.g., an untreated or wild-type sample) and a normalizer (e.g., the expression of a housekeeping gene). In this method, the Ct values for the gene of interest (GOI) in both the test sample(s) and the calibrator sample are normalized in reference to the Ct value of a normalizer (norm) gene obtained from the same two samples. The resulting ΔΔCt value is then utilized to ascertain the fold difference in gene expression.

### Statistical Analysis

Numerous prior studies have already established the sexually dimorphic nature of *cyp19a1a* gene expression in sexually differentiated juvenile and adult fish. Furthermore, various other genes exhibit differing levels of sex-related disparities in their expression, suggesting their potential as reliable indicators of phenotypic sex. However, it was imperative to validate this hypothesis. To this end, our initial examination focused on the expression of *cyp19a1a*, *dmrt1a*, and *dmrt1b* in the gonads of sexually differentiated sea bass at the age of 1.5 years, with their phenotypic sex determined through histological analysis. Subsequently, we assessed sex-related variations in the expression of *cyp19a1a*, *dmrt1a*, and *dmrt1b* in the tail fins of the same fish sample. We employed a randomized completely block design (RCBD) two-way analysis of variance (ANOVA) to evaluate differences in the mean gene expression among sexually differentiated fish, specifically males and females, using the model as described in CoStat (2017):

$$Y_{ijl} = \mu + G_{i} + S_{j} + (GS)_{ij} + e_{ijl}$$

where *Y*_*ijl*_, observation of the *ijl*th parameter measured; *µ*, overall mean; *G*_i_, effect of the *i*th gene; *S*_*j*_, effect of *j*th sex; (*GS*)_*ij*_, interaction genes by sex; *e*_*ijl*_, random error. Significant differences (*P* ≤ 0.05) among means were tested by the method of Duncan (1955). The morphometric characteristics were analyzed by *T*-test.

Furthermore, the final dataset underwent analysis through discriminant analysis (DA). DA is a statistical method that classifies a set of observations into two categories, based on the values of independent continuous categorical variables or predictors, with the categorical variable (sex, male or female in this context) serving as the grouping variable. The outcome of this analysis generates a linear discriminant function, which allocates values to the categorical variable based on the values of the predictor variables (Legendre and Legendre 2012). A discriminant score can be calculated by considering the weighted combination of the predictor variables:

$$D_i=a+b_1x_1+b_2x_2+...+b_nx_n$$

which *D*_*i*_ represents the anticipated score (discriminant score), *x* stands for the predictor, and *b* denotes the discriminant coefficient.

## Results

### Morphometric Characteristics

Total length (TL) and standard length (SL) were significantly different (*p* ≤ 0.05) between females and males where, the mean values between 22.18 ± 0.24 cm, 20.6 ± 0.23 cm, and 20.22 ± 0.32 cm, and 18.43 ± 0.24 cm, respectively. As for body weight (BW) and Fulton’s condition factor (*K*) were no significant differences (*p* ≤ 0.05) between females and males (Table 2).

**Table 2:** Summary statistics for the physical measurements of seabass fish (mean ± SE)

**Parameter**	**Female**	**Male**	***p*-value**
**BW**	100.04 ± 9.5	99.39 ± 6.08	0.955^n.s^
**TL**	22.18 ± 0.24	20.6 ± 0.23	0.001^**^
**SL**	20.22 ± 0.32	18.43 ± 0.24	0.001^**^
**K factor**	0.91 ± 0.105	1.34 ± 0.105	0.055^n.s^

*BW* body weight (g), *TL* total length (cm), *SL* standard length (cm), *K factor* Fulton´s condition factor, *ns* lacking statistical significance

**Significant at a statistical level (*p* ≤ 0.05)

### Expression Profile of (*cyp19a*, *dmrt1a*, and *dmrt1b*) Genes

**Fig. 1:**
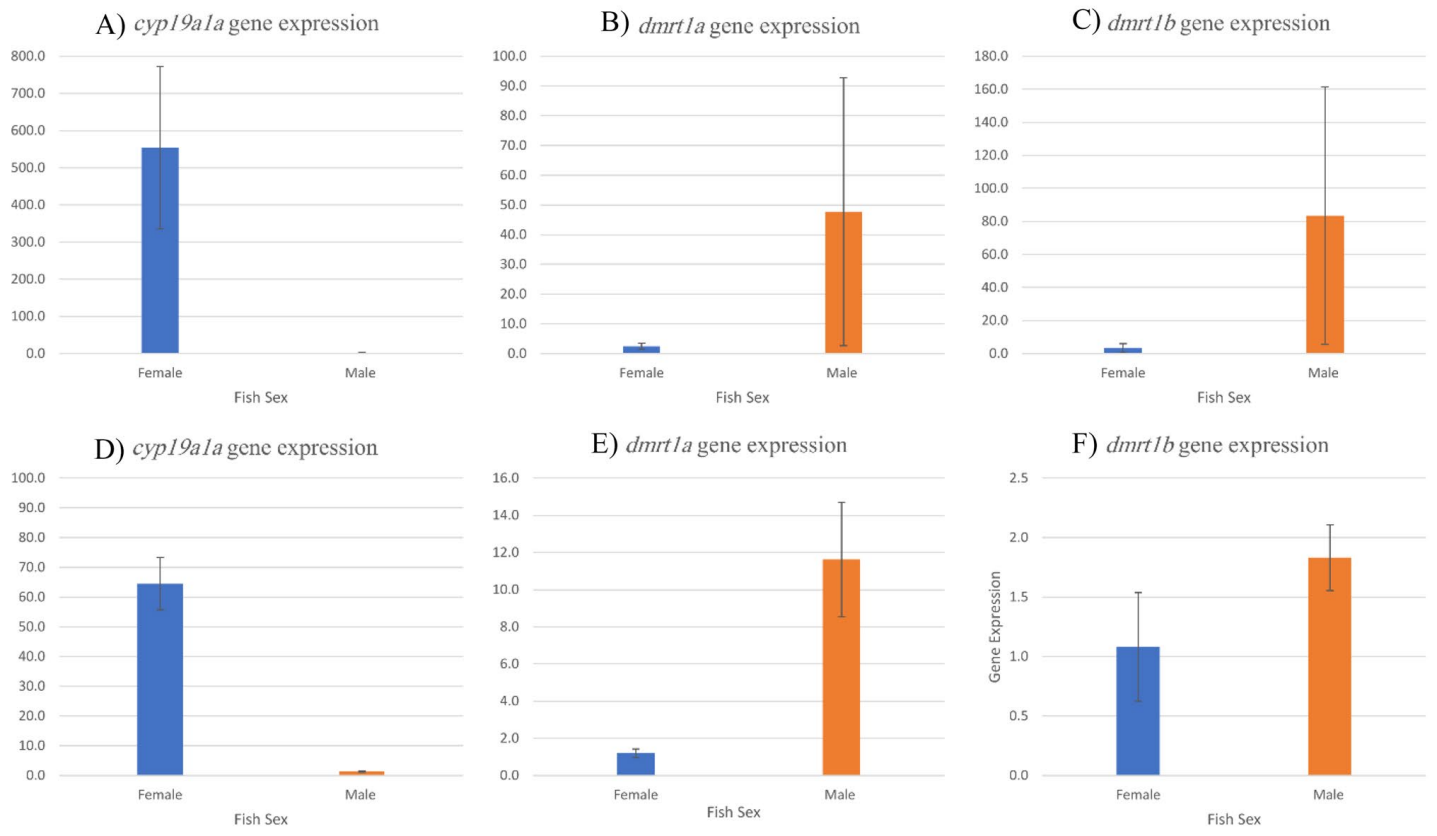
Comparative gene expression of *cyp19a1a* (**A**), *dmrt1a* (**B**), and *dmrt1b* (**C**) in gonads. *cyp19a1a* (**D**), *dmrt1a* (**E**), and *dmrt1b* (**F**) in tail fins tissues. Each value represents mean ± SE

Comparative gene expression analysis of sex determination genes was conducted in tissues of the tail fins as well as the gonads of female and male individuals, investigating that the female ovaries had higher levels of *cyp19a1a* gene expression than the male’s testis. However, in *Dmrt1a* and *Dmrt1b* the gene expression in the male’s testis was higher than female’s ovaries Fig. 1(A–C). The same pattern was detected in tail fin tissues, where *cyp19a1a* gene expression was higher in the female’s tail fins than the males. Additionally, *Dmrt1a* gene expression was higher in males than in females Fig. 1(D–F).

### Sex-Related Differences in Gene Expression of Key Genes in Gonads Tissues

The expression of *cyp19a1a*, *dmrt1a*, and *dmrt1b* in gonads of 1.5-year-old sexually differentiated females and males’ sea bass fish. Analysis of variance II showed significant differences (*p* < 0.05) between gene expression, while between sexes, these differences were insignificant. As shown in Table 3, *cyp19a1a* recorded the highest expression value compared to *dmrt1a* and *dmrt1b* genes. Concerning the effect of sex on gene expression, the results reported that there was no significant difference between males and females. Regarding the interaction between G × S, the results reported that *cyp19a1a* in females’ ovaries recorded a higher value of gene expression than the other interactions.

**Table 3:** The mean of gene expression affected by genes (*cyp19a1a*, *dmrt1a*, and *dmrt1b*), sex (male and female), and their interaction in seabass fish (*D. labrax*) gonads and tail fins tissues

**Factors**		**Tissues**	
**Genes (G)**		**Gonads**	**Tail fins**
*cyp19a1a*		278.07^a^	32.88^a^
*dmrt1a*		25.09^b^	6.4^b^
*dmrt1b*		43.4^b^	1.5^b^
**Sex (S)**			
Female		186.6^a^	22.2^a^
Male		44.5^a^	4.9^b^
**Interaction**			
***G* × *S***			
*cyp19a1a*	Female	554.07^a^	64.4^a^
	Male	2.05^b^	1.34^b^
*dmrt1a*	Female	2.4^b^	1.2^b^
	Male	47.7^b^	11.6^b^
*dmrt1b*	Female	3.28^b^	1.08^b^
	Male	83.5^b^	1.8^b^

Means in the same column (s) followed by the same letter are not significant at a 0.05 probability level

*cyp19a1a* the cytochrome P450 family 19 subfamily A, *dmrt1a* and *dmrt1b* the double sex and mab-3-related transcription factor 1 transcripts

### Sex-Related Differences in Gene Expression of Key Genes in Tail Fin Tissues

The sex-related differences in gene expression of *cyp19a1a*, *dmrt1a*, and *dmrt1b* in tail fins of the same fish samples reported significant differences (*p* < 0.05) between genes and between sex, where *cyp19a1a* was the highest expression value. In terms of sex, females were higher than males in gene expression values. Concerning the interaction between gene and sex, *cyp19a1a* in females’ tail fins had the highest gene expression value among other interactions, as shown in Table 3.

### Define Sex Predictors by Discriminant Analysis (DA)

Discriminant analysis (DA) was obtained to determine which variables including morphometric characteristics (TL, SL, K factor, and body weight (BW)) and all studied genes expression in gonads and tail fins were the best to define sex (males and females).

The DA conducted on morphometric characteristics indicated that TL and SL factors showed significant (*p* ≤ 0.05) indicators for sex differentiation, with *F*-values of 23.011 and 19.870, and Wilks’ *λ* test values of 0.303 and 0.335, enabling a 100% correct classification of fish in both cases. Still, body weight and *K* factor had no significant and high value of Wilks’ *λ* = 1 and 0.68, respectively, as shown in Table 4.

**Table 4:** Assessment of the efficacy and precision in sex determination through individual variables in the discriminant analysis utilizing morphometric parameters

**Variables**	**Wilks’ lambda (*λ*)**	***F*-ratio**	**Males %**	**Females %**	**Total %**	***p*-value**
**BW**	1.000	0.003	0	0	0	1^n.s^
**TL**	0.303	23.011	100	100	100	0.001**
**SL**	0.335	19.870	100	100	100	0.001**
***K*** **factor**	0.680	4.697	83.3	83.3	83.3	0.055^n.s^

*BW* body weight (g), *TL* total length (cm), *SL* standard length (cm), *K factor* Fulton’s condition factor (%), *ns* lacking statistical significance

**Significant at a statistical level (*p* ≤ 0.05)

The expression levels of all studied genes as determined by real-time RT-PCR on samplings from gonads were used to perform the DA. The findings indicate that *cyp19a1a* expression levels played a significant role (*p* ≤ 0.05) in sex differentiation, with an *F*-value of 6.382 and a Wilks’ *λ* test value of 0.610, correctly classifying 75.0% of the fish sex. In contrast, *dmrt1a* and *dmrt1b* did not exhibit significant effects, as indicated by the high value of Wilks’ *λ* = 0.9 (Table 5). While none of the three genes individually achieved a 100% correct classification of fish sex, their collective use with *cyp19a1a* improved classification rates. This underscores the significant role of *cyp19a1a* in discriminating sex in sexually differentiated sea bass.

**Table 5:** Evaluation of the effectiveness and precision in sex determination using individual variables in the discriminant analysis based on gene expression in gonads

**Variables**	**Wilks’ lambda (*λ*)**	***F*-ratio**	**Males %**	**Females %**	**Total %**	***p*-value**
***cyp19a1a*** **(G1)**	0.610	6.382	100	50	75.0	0.030*
***dmrt1a*** **(G2)**	0.908	1.012	16.7	100	58.3	0.338^n.s^
***dmrt1b*** **(G3)**	0.904	1.056	16.7	100	58.3	0.329^n.s^
**G1 G2**			100	66.7	83.3	
**G1 G3**			100	66.7	83.3	
**G1 G2 G3**			100	83.3	91.7	

G1, G2, and G3, genes

*ns* lacking statistical significance

*Significant at a statistical level (*p* ≤ 0.05)

Subsequently, when considering the expression levels of studied genes from tail fin tissues, the results indicated that *cyp19a1a* and *dmrt1a* expression levels significantly (*p* ≤ 0.05) could indicate fish sex, as evidenced by Wilks’ *λ* test values of 0.164 and 0.465, respectively. In contrast, dmrt1b did not exhibit significance, displaying a high Wilks’ *λ* value of 0.835. Notably, *cyp19a1a* emerged as the most influential variable for discriminating between sexes, with a Wilks’* λ* test value of 0.164, successfully classifying 91.7% of the fish (Table 6). Wilks’ *λ* values range from 0 to 1.0, with smaller *λ* values indicating better discrimination among groups. As anticipated, no single variable achieved a 100% correct classification of fish on its own, but when used in conjunction with *cyp19a1a*, classification rates improved. This statistical analysis supports the conclusion that *cyp19a1a* is the most effective indicator for sex determination in sexually differentiated sea bass.

**Table 6:** Assessment of the efficacy and precision in sex determination through individual variables in the discriminant analysis based on tail fin tissue

**Variables**	**Wilks’ lambda (*λ*)**	***F*-ratio**	**Males %**	**Females %**	**Total %**	***p*-value**
*cyp19a1a* (G1)	0.164	51.127	100	83.3	91.7	< 0.000**
*dmrt1a* (G2)	0.465	11.493	83.3	100	91.7	0.007**
*dmrt1b* (G3)	0.835	1.973	66.7	66.7	66.7	0.191^n.s^
G1 G2			100	100	100	
G1 G2 G3			100	100	100	

G1, G2, and G3, genes

*ns* lacking statistical significance

**Significant at a statistical level (*p* ≤ 0.05)

## Discussion

According to size categorizations, Blazquez et al. (1999) have substantiated that female seabass exhibit faster growth rates than their male counterparts right from a young age. This suggests that size can effectively serve as a reliable indicator for sex differentiation in this species. Consequently, employing size grading as a methodology allows us to attribute variations in gene expression and activity to a specific sex during the process of sex differentiation. Our findings are in line with this notion, as they reveal that both total length (TL) and standard length (SL) exhibit significantly greater differences between females and males, further supporting the utility of size-based sex selection in seabass.

In the current study,  the expression of the *cyp19a1a* gene is notably higher in female ovaries compared to other studied genes and aligning with subsequent studies on species such as Southern flounder (Luckenbach et al. 2005), Atlantic halibut (Matsuoka et al. 2006), and rainbow trout (Vizziano et al. 2007). These studies have collectively suggested that *cyp19a1a* gene expression serves as an early indicator of sex differentiation in these species. Additionally, *cyp19a1a* is considered a suitable molecular marker for ovarian differentiation in seabass fish (Piferrer and Guiguen 2008; Blázquez et al. 2008).

The identification of gene expression for all three genes (*cyp19a1a*, *dmrt1a*, and *dmrt1b*) in female and male tail fins, our results indicate that *cyp19a1a* gene expression is the highest in female tail fin tissues, These results may along with a similar scenario in zebrafish which has been documented for SRY HMG box-related gene 9a (*Sox9a*) expression pattern in testis and pectoral fin (Hofsten and Olsson 2005) and with Panagiotopoulou et al. (2023) who found genetic sex markers in Siberian (*Acipenser baerii*) and Atlantic (*A. oxyrinchus*) sturgeons; however, they used a different technique using genetic material extracted from female and male tail fin tissues.

The discriminant analysis finds applications across various research domains, so it is employed in numerical ecology within the context of fisheries management to classify ecosystem exploitation (Tudela et al. 2005). In microbiology, it serves the purpose of discerning the origins of contamination in surface waters, such as whether they stem from human or animal sources (Kaneene et al. 2007). Within the field of forensics, it plays a pivotal role in approximating the gender of unidentified skeletal remains (Kemkes-Grottenthaler 2005). Moreover, discriminant analysis finds applications in medical research, particularly in the differentiation of various types of anemia (Ahluwalia et al. 1995). Notably, it was also employed for the first time to investigate the process of sex differentiation in seabass (Blázquez et al. 2009).

In this study, discriminant analysis was used first to define which variables among morphometric parameters and genes (*cyp19a1a*, *dmrt1a*, and *dmrt1b*) can discriminate fish sex in adult seabass gonads and tail fins. According to the morphometric results, the SL, TL, and *K* factors can correctly classify 100%, 100%, and 91.7% of fish, respectively. This discovery aligns with the established notion that size serves as a reliable marker for sex selection in seabass, as documented by Blazquez et al. (1999). Furthermore, Blázquez et al. (2009) observed that standard length (SL) could accurately classify 75.7% of fish at 330 days post-fertilization (dpf), and this determination was validated through histological examination of gonadal sex.

Based on the results obtained from the gonadal genes, *cyp19a1a* emerged as the most effective predictor for sex differentiation (*F* = 6.382; Wilks’ *λ* test values = 0.610), accurately classifying 75% of the fish on its own. Furthermore, when *cyp19a1a* was combined with other genes (G1, G2, and G3), this combination yielded the highest accuracy, correctly identifying 91.7% of the fish. Additionally, when we applied and validated the discriminant analysis to the same genes in tail fins, our findings consistently showed that *cyp19a1a* alone was the most influential variable in discriminating between sexes (Wilks’ *λ* test = 0.164), accurately classifying 91.7% of the fish. In this case, any combination of variables achieved a 100% accuracy rate. These results corroborate the research conducted by Blázquez et al. (2009), who reported that the *cyp19a1a* gene could perfectly classify 100% of the fish at 330 days post-fertilization (dpf).

## Conclusion

In this work, we concluded that *cyp19a1a* can be used as a genetic marker to discriminate between the fish sexes and as an indicator for ovarian differentiation in sexually differentiated seabass. Additionally, the results reported that tissues from the females’ tail fins had significant levels of *cyp19a1a* gene expression. Therefore, they could be utilized in future studies to distinguish between various adult fish and to identify their sex using molecular markers without killing or sacrificing fish, so they could be used in breeding programs of fish stocks.

## Data Availability

No datasets were generated or analysed during the current study.

## References

[CR1] Ahluwalia N, Lammi-Keefe CJ, Bendel RB (1995). Iron deficiency and anemia of chronic disease in elderly women: a discriminant-analysis approach for differentiation. Am J Clin Nutr.

[CR2] Blazquez M, Carrillo M, Zanuy S, Piferrer F (1999). Sex ratios in offspring of sex-reversed sea bass and the relationship between growth and phenotypic sex differentiation. J Fish Biol.

[CR3] Blázquez M, González A, Papadaki M (2008). Sex-related changes in estrogen receptors and aromatase gene expression and enzymatic activity during early development and sex differentiation in the European sea bass (Dicentrarchus labrax). Gen Comp Endocrinol.

[CR4] Blázquez M, Navarro-Martín L, Piferrer F (2009). Expression profiles of sex differentiation-related genes during ontogenesis in the european sea bass acclimated to two different temperatures. J Exp Zool Part B Mol Dev Evol.

[CR5] Blázquez M, Piferrer F (2004). Cloning, sequence analysis, tissue distribution, and sex-specific expression of the neural form of P450 aromatase in juvenile sea bass (Dicentrarchus labrax). Mol Cell Endocrinol.

[CR6] Blázquez M, Piferrer F (2005). Sea bass (Dicentrarchus labrax) androgen receptor: CDNA cloning, tissue-specific expression, and mRNA levels during early development and sex differentiation. Mol Cell Endocrinol.

[CR7] Brunner B, Hornung U, Shan Z (2001). Genomic organization and expression of the doublesex-related gene cluster in vertebrates and detection of putative regulatory regions for DMRT1. Genomics.

[CR8] Chen J, Zhu Z, Hu W (2022). Progress in research on fish sex determining genes. Water Biol Secur.

[CR9] CoStat 6.451 (2017) Copyright(c) 1998-2017, CoHort Software. http://www.cohort.com

[CR10] Dalla Valle L, Lunardi L, Colombo L, Belvedere P (2002). European sea bass (Dicentrarchus labrax L.) cytochrome P450arom: cDNA cloning, expression and genomic organization. J Steroid Biochem Mol Biol.

[CR11] Deloffre LAM, Martins RST, Mylonas CC, Canario AVM (2009). Alternative transcripts of DMRT1 in the European sea bass: expression during gonadal differentiation. Aquaculture.

[CR12] Duncan DB (1955). Multiple range and multiple F tests. Biometrics.

[CR13] FAO (2019) Fisheries and aquaculture - fisheries and aquaculture - FAO yearbook of fishery and aquaculture statistics. In: FAO. https://www.fao.org/fishery/en/statistics/yearbook. Accessed 21 Mar 2022

[CR14] Gonzalez-Martinez A, De-Pablos-Heredero C, González M (2021). Usefulness of discriminant analysis in the morphometric differentiation of six native freshwater species from Ecuador. Animals.

[CR15] Guan G, Kobayashi T, Nagahama Y (2000). Sexually dimorphic expression of two types of DM (doublesex/Mab-3)-domain genes in a teleost fish, the tilapia (Oreochromis niloticus). Biochem Biophys Res Commun.

[CR16] Guiguen Y, Fostier A, Piferrer F, Chang CF (2010). Ovarian aromatase and estrogens: a pivotal role for gonadal sex differentiation and sex change in fish. Gen Comp Endocrinol.

[CR17] Guo Y, Cheng H, Huang X (2005). Gene structure, multiple alternative splicing, and expression in gonads of zebrafish Dmrt1. Biochem Biophys Res Commun.

[CR18] Halm S, Martínez-Rodríguez G, Rodríguez L (2004). Cloning, characterisation, and expression of three oestrogen receptors (ERα, ERβ1 and ERβ2) in the European sea bass, Dicentrarchus labrax. Mol Cell Endocrinol.

[CR19] Halm S, Rocha A, Miura T (2007). Anti-Müllerian hormone (AMH/AMH) in the European sea bass: Its gene structure, regulatory elements, and the expression of alternatively-spliced isoforms. Gene.

[CR20] Hofsten J, Olsson PE (2005). Zebrafish sex determination and differentiation: involvement of FTZ-F1 genes. Reprod Biol Endocrinol.

[CR21] Kaleem O, Bio Singou Sabi AF (2021). Overview of aquaculture systems in Egypt and Nigeria, prospects, potentials, and constraints. Aquac Fish.

[CR22] Kaneene JB, Miller RA, Sayan R (2007). Considerations when using discriminant function analysis of antimicrobial resistance profiles to identify sources of fecal contamination of surface water in Michigan. Appl Environ Microbiol.

[CR23] Kemkes-Grottenthaler A (2005). Sex determination by discriminant analysis: an evaluation of the reliability of patella measurements. Forensic Sci Int.

[CR24] Legendre P, Legendre L (2012). Numerical ecology - P.

[CR25] Lin A, Xiao S, Xu S (2017). Identification of a male-specific DNA marker in the large yellow croaker (Larimichthys crocea). Aquaculture.

[CR26] Livak KJ, Schmittgen TD (2001). Analysis of relative gene expression data using real-time quantitative PCR and the 2−ΔΔCT method. Methods.

[CR27] Long J, Zheng S, Wang X (2020). Role of TGF-β signaling pathway in sex determination and differentiation in fish. J Fish China.

[CR28] Luckenbach JA, Early LW, Rowe AH (2005). Aromatase cytochrome P450: cloning, intron variation, and ontogeny of gene expression in southern flounder (Paralichthys lethostigma). J Exp Zool Part A Comp Exp Biol.

[CR29] Marchand O, Govoroun M, D’Cotta H (2000). DMRT1 expression during gonadal differentiation and spermatogenesis in the rainbow trout, Oncorhynchus mykiss. Biochim Biophys Acta - Gene Struct Expr.

[CR30] Martins RST, Deloffre LAM, Mylonas CC (2007). Developmental expression of DAX1 in the European sea bass, Dicentrarchus labrax: lack of evidence for sexual dimorphism during sex differentiation. Reprod Biol Endocrinol.

[CR31] Matsuoka MP, van Nes S, Andersen Ø (2006). Real-time PCR analysis of ovary- and brain-type aromatase gene expression during Atlantic halibut (Hippoglossus hippoglossus) development. Comp Biochem Physiol Part B Biochem Mol Biol.

[CR32] Panagiotopoulou H, Marzecki K, Gawor J (2023). Extensive search of genetic sex markers in Siberian (Acipenser baerii) and Atlantic (A. oxyrinchus) sturgeons. Aquaculture.

[CR33] Piferrer F, Blázquez M, Navarro L, González A (2005). Genetic, endocrine, and environmental components of sex determination and differentiation in the European sea bass (Dicentrarchus labrax L.). Gen Comp Endocrinol.

[CR34] Piferrer F, Guiguen Y (2008). Fish gonadogenesis. part II: molecular biology and genomics of sex differentiation. Rev Fish Sci.

[CR35] Saillant E, Chatain B, Menu B (2003). Sexual differentiation and juvenile intersexuality in the European sea bass (Dicentrarchus labrax). J Zool.

[CR36] Socorro S, Martins RS, Deloffre L (2007). A cDNA for European sea bass (Dicentrachus labrax) 11β-hydroxylase: gene expression during the thermosensitive period and gonadogenesis. Gen Comp Endocrinol.

[CR37] Tudela S, Coll M, Palomera I (2005). Developing an operational reference framework for fisheries management on the basis of a two-dimensional index of ecosystem impact Abstract ICES. Journal of Marine Science.

[CR38] Vandeputte M, Dupont-Nivet M, Chavanne H, Chatain B (2007). A polygenic hypothesis for sex determination in the European sea bass Dicentrarchus labrax. Genetics.

[CR39] Vizziano D, Randuineau G, Baron D (2007). Characterization of early molecular sex differentiation in rainbow trout, Oncorhynchus mykiss. Dev Dyn.

[CR40] Wootton, R.J. and Smith, C. (2014). Sex determination. In Reproductive Biology of Teleost Fishes (eds R.J. Wootton and C. Smith). 10.1002/9781118891360.CH2

[CR41] Yamaguchi A, Lee KH, Fujimoto H (2006). Expression of the DMRT gene and its roles in early gonadal development of the Japanese pufferfish Takifugu rubripes. Comp Biochem Physiol Part D Genomics Proteomics.

